# Protective Effects of Taraxasterol against Ethanol-Induced Liver Injury by Regulating CYP2E1/Nrf2/HO-1 and NF-*κ*B Signaling Pathways in Mice

**DOI:** 10.1155/2018/8284107

**Published:** 2018-09-23

**Authors:** Lu Xu, Yifan Yu, Rui Sang, Jinxia Li, Bingjie Ge, Xuemei Zhang

**Affiliations:** Department of Animal Medicine, Agricultural College of Yanbian University, Gongyuan Street, Yanji, Jilin 133002, China

## Abstract

Taraxasterol, a pentacyclic-triterpene compound, is one of the main active components isolated from the traditional Chinese medicinal herb *Taraxacum*. The objective of this study is to evaluate the protective effects of taraxasterol and its possible underlying mechanisms against ethanol-induced liver injury in mice. ICR mice were fed with Lieber-DeCarli diet containing 5% ethanol for 10 d and then challenged with a single dose of 20% ethanol (5 g/kg BW) by intragastric administration. The mice were intragastrically treated daily with taraxasterol (2.5, 5, and 10 mg/kg). Tiopronin was used as a positive control. The liver index was calculated, and the levels of alanine aminotransferase (ALT), aspartate aminotransferase (AST), triglyceride (TG), tumor necrosis factor-*α* (TNF-*α*), and interleukin-6 (IL-6) in sera were detected. The contents of reactive oxygen species (ROS), malondialdehyde (MDA), and glutathione (GSH) and the activity of superoxide dismutase (SOD) in the livers were measured. The histopathological changes of liver tissues were observed by hematoxylin and eosin (H&E) staining. The protein expression levels of hepatic cytochrome P450 2E1 (CYP2E1), nuclear factor erythroid 2-related factor 2 (Nrf2), antioxidant protein heme oxygenase-1 (HO-1), and nuclear factor-kappa B (NF-*κ*B) signaling pathway in liver tissues were detected by immunohistochemistry and Western blot methods. Taraxasterol significantly reduced the ethanol-induced increases of liver index, ALT, AST, and TG levels in sera and TG and MDA contents in the livers and hepatic ROS production and suppressed the ethanol-induced decreases of hepatic GSH level and SOD activity. Taraxasterol also significantly inhibited the secretion of proinflammatory cytokines TNF-*α* and IL-6 induced by ethanol. In addition, taraxasterol improved the liver histopathological changes in mice with ethanol-induced liver injury. Further studies revealed that taraxasterol significantly inhibited the ethanol-induced upregulation of CYP2E1, increased the ethanol-induced downregulation of Nrf2 and HO-1, and inhibited the degradation of inhibitory kappa B*α* (I*κ*B*α*) and the expression level of NF-*κ*B p65 in liver tissues of ethanol-induced mice. These findings suggest that taraxasterol possesses the potential protective effects against ethanol-induced liver injury in mice by exerting antioxidative stress and anti-inflammatory response via CYP2E1/Nrf2/HO-1 and NF-*κ*B signaling pathways.

## 1. Introduction

Due to drinking habits and alcohol abuse, the morbidity and mortality of alcoholic liver disease (ALD) are increasing worldwide. ALD has become one of the most important health problems all over the world, and it is also the leading cause of liver disease deaths [1, 2]. At present, oxidative stress and inflammation are considered to be the two main mechanisms involved in the pathogenesis of ALD [3]. Ethanol induces the oxidation of hepatic cytochrome P450 2E1 (CYP2E1) enzyme, and the oxidation of CYP2E1 results in the overproduction of reactive oxygen species (ROS), such as hydroxyl radical, hydrogen peroxide, and superoxide anion. These metabolites can inhibit or deplete endogenous enzymatic and nonenzymatic antioxidants, cause oxidative stress, and lead to liver cell apoptosis and liver lipid peroxidation [4]. In addition, ethanol and its metabolites can increase the release of proinflammatory cytokines such as tumor necrosis factor-*α* (TNF-*α*) and interleukin-6 (IL-6). Excessive release of these inflammatory cytokines leads to the imbalance of cytokines and immune dysfunction, which further damage liver function. The release of these inflammatory cytokines is closely related to the activation of nuclear factor-kappa B (NF-*κ*B) inflammatory signaling pathway, which plays an important role in ethanol-induced ALD [5]. Therefore, antioxidative and anti-inflammatory agents are considered to be potential therapeutic drugs against liver injury induced by ethanol [6].

The cornerstone of prevention and treatment for alcoholic liver injury is abstinence from alcohol. Currently, other main treatments are pharmacological therapies, some antioxidant and anti-inflammatory drugs such as corticosteroids, glutathione, pentoxifylline, colchicine, and S-adenosyl methionine may be beneficial to patients with ALD, but these treatments are accompanied by the side effects such as renal impairment, jaundice, etc. [7, 8]. Accordingly, great attention has been paid to natural herbal plants and their active ingredients as new therapeutic drugs against ALD due to their high efficiency, few side effects, and low cost.

*Taraxacum* (dandelion) is the whole herb of *Taraxacum sinicum* Kitag, *Taraxacum mongolicum* Hand.-Mazz, or same genus plants (composite). It has long been widely used in traditional oriental food and medicine for its free radical scavenging, anti-inflammatory, antiseptic, antioxidation, antitumor, antiatherosclerosis, and hepatoprotective properties [9–11]. Especially, the hepatoprotective effects of *Taraxacum* against alcohol and acetaminophen-induced oxidative stress have been reported [12, 13]. However, its antioxidant and hepatoprotective constituents and modalities of action have not been issued. Taraxasterol, a pentacyclic-triterpene, is one of the main active constituents isolated from *Taraxacum*. Moreover, recent studies have shown that natural terpene compounds in Chinese herbal medicine can delay and reduce alcoholic liver damage [14]. Our studies have demonstrated that taraxasterol has the *in vitro* anti-inflammatory activity by suppressing the production of various cytokines and inflammatory mediators in LPS-induced murine RAW 264.7 macrophages [15, 16]. We have also reported that taraxasterol has the *in vivo* protective effects on ovalbumin-induced allergic asthma [17], LPS-induced endotoxic shock in mice [18], and adjuvant-induced arthritis in rats [19]. However, no study thus far has issued whether taraxasterol possesses protective effects against alcoholic liver injury and what the underlying mechanisms. Therefore, as a part of our ongoing screening program to evaluate the antioxidative and anti-inflammatory potentials of natural compounds, the objective of this present study is to explore the protective effects of taraxasterol and its possible underlying mechanisms on alcoholic liver injury in mice.

## 2. Materials and Methods

### 2.1. Animals

Male ICR mice (20 ± 2 g) were purchased from Changchun Yisi Experiment Animals Co. Ltd. (Certificate no. SCXK (J) 2003-0008, Changchun, Jilin, China). The mice were fed in the standard experimental conditions (room temperature 23 ± 1°C, relative humidity 55 ± 5%) with a 12 h light/dark cycle and received food and water ad libitum. Before the experiment, the mice were adapted to the experimental environment for 1 week. All animal experimental procedures were carried out in accordance with the guidelines of the Animal Care Committee of Yanbian University (Certificate no. SCXK (J) 2011-0007, Yanji, Jilin, China).

### 2.2. Drugs and Reagents

Taraxasterol was supplied from Chengdu Fenruisi Biotechnology Co. Ltd. (Chengdu, Sichuan, China), and its purity was 99.5% based on HPLC analysis. Tiopronin (TPN, no. 036140404) was purchased from Xinyi Pharmaceutical Co. Ltd. (Xinyi, Henan, China). Absolute ethanol was purchased from Sigma-Aldrich (St. Louis, MO, USA). Liber-DeCarli liquid diet was obtained from Tropic Animal Feed High-Tech Co. Ltd. (Nantong, Jiangsu, China). Alanine aminotransferase (ALT), aspartate aminotransferase (AST), triglyceride (TG), reactive oxygen species (ROS), malondialdehyde (MDA), glutathione (GSH), and superoxide dismutase (SOD) commercial reagent kits were purchased from Nanjing Jiancheng Bioengineering Institute (Nanjing, Jiangsu, China). Mouse TNF-*α* and IL-6 ELISA kits were purchased from BioLegend Inc. (San Diego, CA, USA). Anti-CYP2E1, I kappa B*α* (I*κ*B*α*), nuclear factor-*κ*B (NF-*κ*B) p65, nuclear factor erythroid 2-related factor 2 (Nrf2), and heme oxygenase-1 (HO-1) antibodies were purchased from Cell Signaling Technology Inc. (Danvers, MA, USA). *β*-Actin, horseradish peroxidase- (HRP-) conjugated goat anti-mouse IgG, and goat anti-rabbit IgG were purchased from Santa Cruz (Santa Cruz, CA, USA). Diaminobenzidine (DAB), bicinchoninic acid (BCA), and chemiluminescent (ECL) reagent kits were purchased from Beyotime Biotech. (Shanghai, China).

### 2.3. Establishment of Mouse Ethanol-Induced Liver Injury and Drug Treatment

The model of ethanol-induced liver injury in mice was established as previously described [20]. The dose of taraxasterol used in this experiment was based on our previous studies [17–19]. The mice were randomly divided into six groups (*n* = 10): normal group, ethanol (EtOH) group, taraxasterol (2.5, 5, and 10 mg/kg, respectively) groups, and TPN group. All mice were fed with control Lieber-DeCarli diet for 5 d in order to acclimatize to liquid diet. From day 6, except for the normal group, all mice were fed with Lieber-DeCarli diet containing 5% (*v/v*) ethanol for 10 d. Meantime, the mice in the taraxasterol groups were administered intragastrically with taraxasterol at doses of 2.5, 5, and 10 mg/kg, respectively, in 0.5% carboxymethyl cellulose sodium (CMC-Na) once per day for 10 d. The mice in the TPN group were orally treated with 20 mg/kg of TPN as a positive control. The mice in the normal and EtOH groups were orally treated with equal volume of 0.5% CMC-Na. On day 16, the mice in the EtOH and drug treatment groups were gavaged with a single dose of 20% (*v/v*) ethanol (5 g/kg BW) in the early morning, whereas the mice in the normal group were gavaged with isocaloric maltose dextrin. Nine hours later, the mice were weighed and anesthetized by the inhalation of diethyl ether, and the blood samples were collected from retro-orbital plexus. The mice were then sacrificed by cervical dislocation, and the liver tissues were quickly removed. All blood samples and liver tissues were used to detect the following series of indicators.

### 2.4. Determination of Liver Index of Mice

Nine hours after the last ethanol challenge, the body weight of mice was measured. The liver was removed, and the wet weight of the liver was weighed. The liver index was calculated by the following formula: liver index (%) = liver wet weight/mouse body weight × 100%.

### 2.5. Determination of Serum ALT, AST, and TG in Mice

Blood was collected and serum was separated by centrifugation (4°C, 3000 rpm, 10 min). The levels of serum ALT, AST, and TG were determined according to the requirements of the instructions provided in reagent kits. The values were expressed as U/l or mmol/l of sera.

### 2.6. Determination of ROS Production, MDA and GSH Levels, SOD Activity, and TG Content in the Liver of Mice

Liver tissue was homogenized in PBS to prepare 10% liver homogenate, then centrifugated (4°C, 3000 rpm, 10 min), and the supernatant was collected. The production of ROS, the levels of MDA and GSH, the activity of SOD, and the content of TG in the supernatant of liver homogenate were determined according to the requirements of the instructions provided in reagent kits. The protein concentration in the liver was measured with BCA protein assay reagent kit. The values were normalized to total protein in liver tissue. ROS production was expressed as % of normal, MDA level was expressed as nmol/mg of protein, GSH level was expressed as *μ*mol/g of protein, SOD activity was expressed as U/mg of protein, and TG level was expressed as mmol/g of protein.

### 2.7. Determination of Serum TNF-*α* and IL-6 in Mice

Blood was collected, and serum was separated by centrifugation (4°C, 3000 rpm, 10 min). The levels of serum TNF-*α* and IL-6 were determined by sandwich ELISA method according to the requirements of the instructions provided in reagent kits. The values were expressed as pg/ml of sera based on appropriate standard curve.

### 2.8. Histopathological Observation of Liver Tissues

The liver tissues of mice were fixed in 10% neutral phosphate-buffered formalin solution, then dehydrated and embedded in paraffin to make conventional paraffin sections. The sections were cut into 4 *μ*m thickness and stained with hematoxylin and eosin (H&E). The histopathological changes of liver tissues were observed under the optical microscope.

### 2.9. Immunohistochemical Analysis of CYP2E1 Expression in Liver Tissues of Mice

The expression of CYP2E1 in liver tissues of mice was detected by immunohistochemistry. The liver tissues were fixed, embedded, and sliced into 4 *μ*m thickness sections. The slices were deparaffinized and hydrated, and then incubated with CYP2E1 antibody for overnight at 4°C, followed by the antibiotin detection kit and DAB staining and hematoxylin staining. The expression of CYP2E1 protein in liver tissues was detected by chromogenic reaction under light microscope.

### 2.10. Western Blot Analysis of CYP2E1, Nrf2, HO-1, I*κ*B*α*, and p65 Expressions in Liver Tissues of Mice

The liver tissues were homogenized, lysed, and centrifuged (4°C, 12000 g, 10 min). The protein concentration was determined by BCA protein assay reagent kit. The equal amounts of protein were separated on 10% SDS-PAGE gel electrophoresis, then the target protein strip was transferred to the PVDF membrane. The membrane was incubated with primary antibodies anti-CYP2E1, Nrf2, HO-1, I*κ*B*α*, NF-*κ*B p65, and *β*-actin diluted in 5% skim milk in Tween 20/Tris-buffered saline (TTBS) for overnight at 4°C. The membrane was washed with TTBS and incubated with HRP-conjugated secondary antibodies for 1 h at room temperature. The membrane was washed with TTBS again and exposed to ECL solution. The image was developed using gel imager. The light density of the target protein band was analyzed with Quantity One analysis software, and the relative expression of the target protein was normalized to *β*-actin.

### 2.11. Statistical Analysis

All values are expressed as the means ± SEMs. One-way analysis of variance (ANOVA) and Student's *t*-test were used to assess the differences between the groups. The statistical analyses were conducted using SPSS 17.0 software (SPSS, China). A *P* value < 0.05 was considered significant.

## 3. Results

### 3.1. Effects of Taraxasterol on Liver Index in Mice with Ethanol-Induced Liver Injury

**Figure 1:**
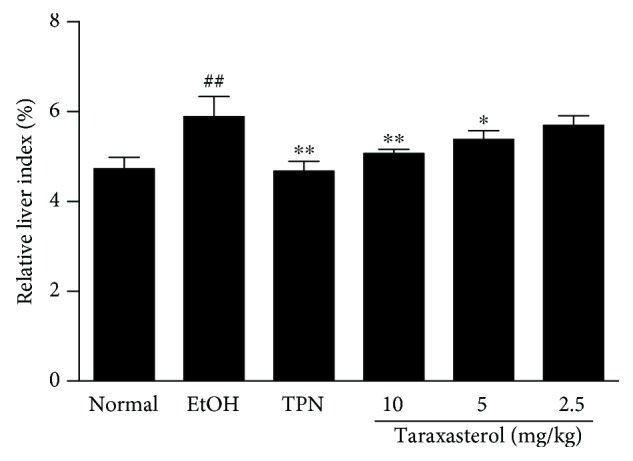
Effects of taraxasterol on liver index in mice with ethanol-induced liver injury. The mice were treated with taraxasterol (2.5, 5, and 10 mg/kg, respectively) or TPN and induced with EtOH as described in Materials and Methods. The liver was removed and weighed, and the liver index was calculated. The values are expressed as the means ± SEMs. ^##^*P* < 0.01 vs. normal group; ^∗^*P* < 0.05 and ^∗∗^*P* < 0.01 vs. EtOH group.

The effect of taraxasterol on ethanol-induced liver index was firstly evaluated. As shown in Figure 1, the liver index of mice in the EtOH group significantly increased compared with the normal group (*P* < 0.01). Treatment with taraxasterol at 5 and 10 mg/kg significantly reduced the ethanol-induced increase of liver index compared with the EtOH group (*P* < 0.05 or *P* < 0.01). The liver index in 2.5 mg/kg of the taraxasterol group also indicated a decreasing trend, but there was no significant difference compared with the EtOH group (*P* > 0.05). The results showed that taraxasterol could dose dependently reduce the increase of liver index in mice with ethanol-induced liver injury.

### 3.2. Effects of Taraxasterol on the Levels of Serum AST and ALT in Mice with Ethanol-Induced Liver Injury

**Figure 2:**
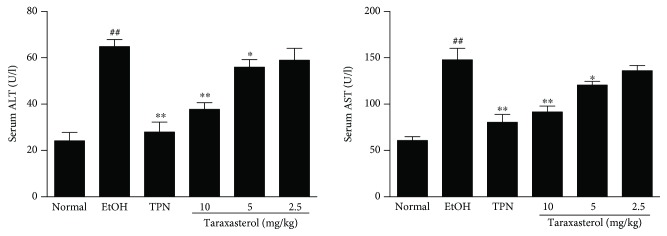
Effects of taraxasterol on the levels of serum ALT and AST in mice with ethanol-induced liver injury. The mice were treated with taraxasterol (2.5, 5, and 10 mg/kg, respectively) or TPN and induced with EtOH as described in Materials and Methods. The levels of serum ALT and AST were determined by commercial reagent kits. The values represent the means ± SEMs and are expressed as U/l of sera. ^##^*P* < 0.01 vs. normal group; ^∗^*P* < 0.05 and ^∗∗^*P* < 0.01 vs. EtOH group.

To assess the effect of taraxasterol on liver function, the levels of serum biomarker AST and ALT were tested. As shown in Figure 2, the levels of serum AST and ALT significantly raised in the EtOH group compared with the normal group (*P* < 0.01). Treatment with taraxasterol at 5 and 10 mg/kg significantly suppressed the ethanol-induced increase of serum AST and ALT levels compared with the EtOH group (*P* < 0.05 or *P* < 0.01), although there was no obvious difference between 2.5 mg/kg of the taraxasterol group and the EtOH group (*P* > 0.05). The results showed that taraxasterol could inhibit the increase of serum AST and ALT levels in mice with ethanol-induced liver injury in a dose-dependent manner.

### 3.3. Effects of Taraxasterol on the Contents of Serum and Hepatic TG in Mice with Ethanol-Induced Liver Injury

**Figure 3:**
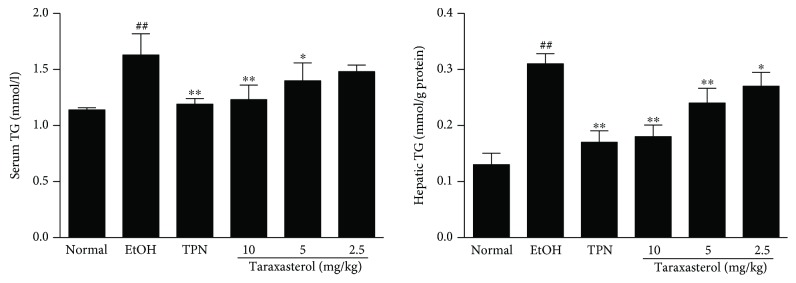
Effects of taraxasterol on the contents of TG in sera and liver tissues in mice with ethanol-induced liver injury. The mice were treated with taraxasterol (2.5, 5, and 10 mg/kg, respectively) or TPN and induced with EtOH as described in Materials and Methods. The contents of TG in sera and liver tissues were determined by commercial reagent kits. The values represent the means ± SEMs and are expressed as mmol/l of sera and mmol/g of liver protein. ^##^*P* < 0.01 vs. normal group; ^∗^*P* < 0.05 and ^∗∗^*P* < 0.01 vs. EtOH group.

To quantitatively confirm the effect of taraxasterol on ethanol-induced lipid accumulation, the contents of TG in sera and liver tissues were measured. As shown in Figure 3, the contents of TG in sera and liver tissues significantly raised in the EtOH group compared with the normal group (*P* < 0.01). Treatment with taraxasterol at 5 and 10 mg/kg significantly reduced the ethanol-induced increase of serum TG content compared with the EtOH group (*P* < 0.05 or *P* < 0.01). Taraxasterol at 2.5, 5, and 10 mg/kg significantly reduced the ethanol-induced increase of TG content in liver tissues compared with the EtOH group in a dose-dependent manner (*P* < 0.05 or *P* < 0.01). The results showed that taraxasterol could decrease the contents of TG and inhibited hepatic lipid accumulation in mice with ethanol-induced liver injury.

### 3.4. Effects of Taraxasterol on the Production of ROS, the Levels of MDA and GSH, and the Activity of SOD in Liver Tissues of Mice with Ethanol-Induced Liver Injury

**Figure 4:**
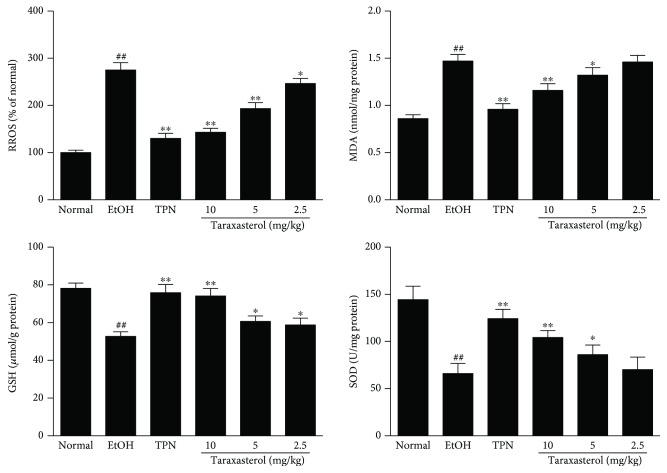
Effects of taraxasterol on the production of ROS, the levels of MDA and GSH, and the activity of SOD in liver tissues of mice with ethanol-induced liver injury. The mice were treated with taraxasterol (2.5, 5, and 10 mg/kg, respectively) or TPN and induced with EtOH as described in Materials and Methods. The production of ROS, the levels of MDA and GSH, and the activity of SOD in liver tissues were determined by commercial reagent kits. The values represent the means ± SEMs and are expressed as % of normal, nmol/mg of protein, *μ*mol/g of protein, and U/mg of protein, respectively. ^##^*P* < 0.01 vs. normal group; ^∗^*P* < 0.05 and ^∗∗^*P* < 0.01 vs. EtOH group.

To test the effects of taraxasterol against oxidative stress induced by ethanol, the production of ROS, the levels of MDA and GSH, and the activity of SOD in liver tissues were measured. As shown in Figure 4, ROS production and MDA level were significantly increased, and GSH level and SOD activity were significantly decreased in the liver tissues of the EtOH group compared with the normal group (*P* < 0.01). Taraxasterol at 2.5, 5, and 10 mg/kg significantly decreased ROS production compared with the EtOH group (*P* < 0.05 or *P* < 0.01). Taraxasterol at 5 and 10 mg/kg significantly reduced the ethanol-induced increase of MDA content compared with the EtOH group (*P* < 0.05 or *P* < 0.01). Taraxasterol at 2.5, 5, and 10 mg/kg significantly suppressed the ethanol-induced decrease of GSH level compared with the EtOH group (*P* < 0.05 or *P* < 0.01). Taraxasterol at 5 and 10 mg/kg significantly suppressed ethanol-induced reduction of SOD activity compared with the EtOH group (*P* < 0.05 or *P* < 0.01). The results showed that taraxasterol could decrease ROS production and MDA level and improve GSH level and SOD activity in liver tissues of mice with ethanol-induced liver injury in a dose-dependent manner.

### 3.5. Effects of Taraxasterol on the Production of Serum TNF-*α* and IL-6 in Mice with Ethanol-Induced Liver Injury

**Figure 5:**
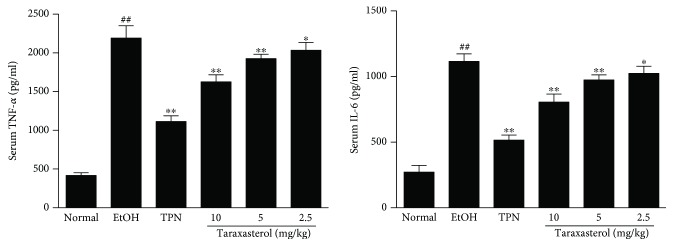
Effects of taraxasterol on the production of serum TNF-*α* and IL-6 in mice with ethanol-induced liver injury. The mice were treated with taraxasterol (2.5, 5, and 10 mg/kg, respectively) or TPN and induced with EtOH as described in Materials and Methods. The levels of serum TNF-*α* and IL-6 were determined by ELISA kits. The values represent the means ± SEMs and are expressed as pg/ml of sera. ^##^*P* < 0.01 vs. normal group; ^∗^*P* < 0.05 and ^∗∗^*P* < 0.01 vs. EtOH group.

To evaluate the anti-inflammatory activity of taraxasterol induced by ethanol, two main pro-inflammatory cytokines TNF-*α* and IL-6 levels were determined by ELISA. As shown in Figure 5, serum TNF-*α* and IL-6 levels were significantly increased in the EtOH group compared with the normal group (*P* < 0.01). However, treatment with taraxasterol at 2.5, 5, and 10 mg/kg significantly inhibited ethanol-induced TNF-*α* and IL-6 production compared with the EtOH group in a dose-dependent manner (*P* < 0.05 or *P* < 0.01). The results showed that taraxasterol could inhibit inflammatory cytokines TNF-*α* and IL-6 production in mice with ethanol-induced liver injury.

### 3.6. Effects of Taraxasterol on Liver Histopathology in Mice with Ethanol-Induced Liver Injury

**Figure 6:**
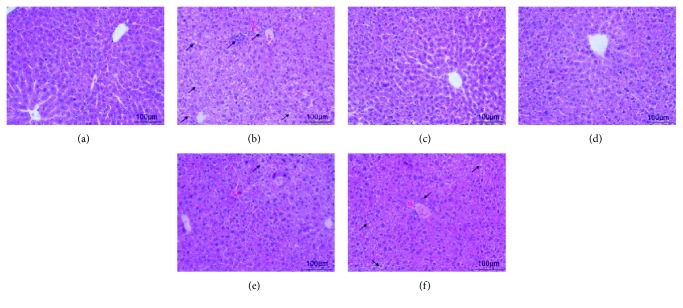
Effects of taraxasterol on liver histopathology in mice with ethanol-induced liver injury (200x). The mice were treated with taraxasterol (2.5, 5, and 10 mg/kg, respectively) or TPN and induced with EtOH as described in Materials and Methods. The histopathological changes of liver tissues were observed under the optical microscope by H&E staining. (a) Normal group; (b) EtOH group; (c) TPN group; (d) 10 mg/kg of taraxasterol group; (e) 5 mg/kg of taraxasterol group; (f) 2.5 mg/kg of taraxasterol group.

To directly evaluate the protective effects of taraxasterol on ethanol-induced liver injury, liver histopathological observation was also performed. As shown in Figure 6, the liver tissues were intact, the hepatic lobules were clear, and the hepatocytes were regularly arranged in the normal group (Figure 6(a)). In the EtOH group, it was found that the basic architecture of liver cells was disappeared, which displayed apparent steatosis, inflammatory cell infiltration, and liver cell swelling (Figure 6(b)). In contrast, treatment with taraxasterol significantly ameliorated the degree of liver histopathological alterations, especially in TNP and 10 mg/kg of taraxasterol groups (Figures 6(c) and 6(d)). Only slight fatty degenerations were found in 5 mg/kg and 2.5 mg/kg of the taraxasterol groups (Figures 6(e) and 6(f)).

### 3.7. Effects of Taraxasterol on the Expression of CYP2E1 in Liver Tissues of Mice with Ethanol-Induced Liver Injury

**Figure 7:**
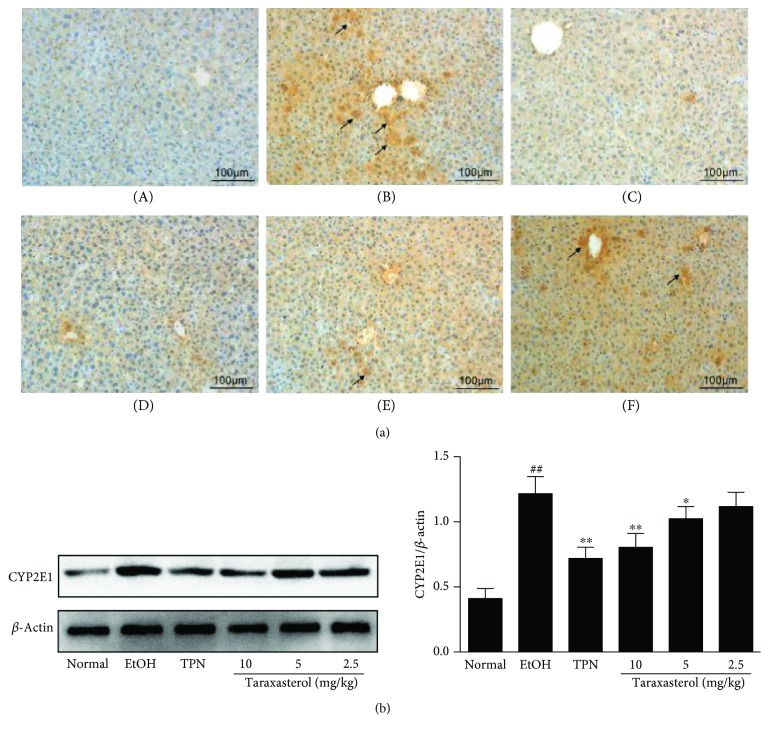
Effects of taraxasterol on the expression of CYP2E1 in liver tissues of mice with ethanol-induced liver injury. The mice were treated with taraxasterol (2.5, 5, and 10 mg/kg, respectively) or TPN and induced with EtOH as described in Materials and Methods. (a) The expression of CYP2E1 in liver tissues was examined by immunohistochemical analysis (200x, brown yellow granules indicate positive reaction). (A) Normal group; (B) EtOH group; (C) TPN group; (D) 10 mg/kg of taraxasterol group; (E) 5 mg/kg of taraxasterol group; (F) 2.5 mg/kg of taraxasterol group. (b) The expression of CYP2E1 in liver tissues was examined by western blot analysis. The relative expression of CYP2E1 protein was normalized to *β*-actin. The values represent the means ± SEMs. ^##^*P* < 0.01 vs. normal group; ^∗^*P* < 0.05 and ^∗∗^*P* < 0.01 vs. EtOH group.

CYP2E1 is a major contributor to ROS production, so we examined the expression of CYP2E1 in liver tissues by immunohistochemical and Western blot analyses. As shown in Figure 7(a), immunohistochemical analysis indicated that there was increased CYP2E1 expression in the EtOH group compared with the normal group (brown yellow granules indicated positive reaction). However, treatment with taraxasterol inhibited the ethanol-induced upregulation of CYP2E1 expression at different degrees compared with the EtOH group, especially in 10 mg/kg and 5 mg/kg of the taraxasterol groups. As shown in Figure 7(b), Western blot analysis also indicated that taraxasterol at 5 and 10 mg/kg significantly inhibited the ethanol-induced upregulation of CYP2E1 expression (*P* < 0.05 or *P* < 0.01), and it consists of the result of immunohistochemical analysis. It showed that taraxasterol could exert antioxidative stress by inhibiting CYP2E1 expression in liver tissues of mice with ethanol-induced liver injury.

### 3.8. Effects of Taraxasterol on the Expressions of Nrf2, HO-1, I*κ*B*α*, and NF-*κ*B p65 in Liver Tissues of Mice with Ethanol-Induced Liver Injury

**Figure 8:**
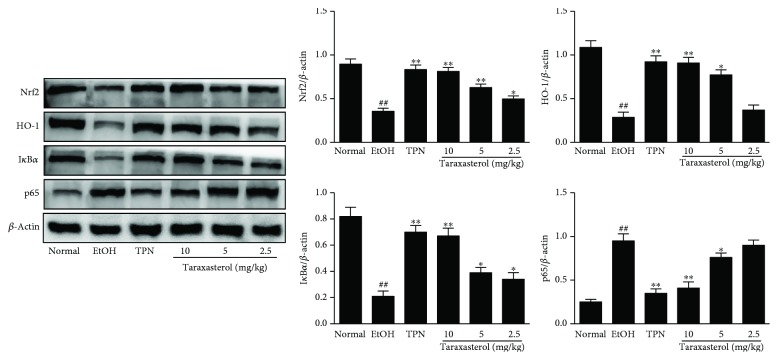
Effects of taraxasterol on the expressions of Nrf2, HO-1, I*κ*B*α*, and NF-*κ*B p65 in liver tissues of mice with ethanol-induced liver injury. The mice were treated with taraxasterol (2.5, 5, and 10 mg/kg, respectively) or TPN and induced with EtOH as described in Materials and Methods. The expressions of Nrf2, HO-1, I*κ*B*α*, and NF-*κ*B p65 in liver tissues were measured by Western blot analysis. The relative expressions of these proteins were normalized to *β*-actin. The values represent the means ± SEMs. ^##^*P* < 0.01 vs. normal group; ^∗^*P* < 0.05 and ^∗∗^*P* < 0.01 vs. EtOH group.

To further examine the effects of taraxasterol on oxidant defense system and inflammatory signaling pathway, the expressions of Nrf2, HO-1, I*κ*B*α*, and NF-*κ*B p65 in liver tissues were determined by Western blot. As shown in Figure 8, the expressions of Nrf2, HO-1, and I*κ*B*α* proteins in the EtOH group were significantly decreased, and the expression of NF-*κ*B p65 protein in the EtOH group was increased compared with the normal group (*P* < 0.01). In contrast, treatment with taraxasterol significantly increased the ethanol-induced downregulation of Nrf2 and HO-1 proteins (*P* < 0.05 or *P* < 0.01) and inhibited the degradation of I*κ*B*α* protein and the expression of NF-*κ*B p65 protein compared with the EtOH group in a dose-dependent manner (*P* < 0.05 or *P* < 0.01). It showed that taraxasterol could enhance the oxidant defense system by upregulating Nrf2/HO-1 proteins and suppress inflammation by regulating I*κ*B*α*/NF-*κ*B p65 proteins of NF-*κ*B pathway.

## 4. Discussion

Alcoholic liver disease is a liver disease caused by excessive drinking in the long term. It is associated with oxidative stress, fatty degeneration, and inflammation. The liver is the main organ of alcohol metabolism and the target organ of alcohol injury. Currently, a chronic-plus-single-binge ethanol-feeding mouse model is widely and frequently used for alcoholic liver injury. This model is close to the drinking behaviors in human, which simulates the pathogenesis of alcoholic liver injury in clinical patients [20]. Therefore, in the present study, we established the chronic-plus-single-binge ethanol-feeding model in mice and explored the protective effects and underlying mechanisms against ethanol-induced liver injury. Our results demonstrate that taraxasterol has the potential protective effects against ethanol-induced liver injury in mice by exerting antioxidative stress and anti-inflammatory response via CYP2E1/Nrf2/HO-1 and NF-*κ*B signaling pathways.

ALT and AST are the enzymes that catalyze the amino transfer between amino acids and ketoacids, mainly localize in the liver cells. When the hepatocytes are injured, the permeability of the cell membrane increases, and ALT and AST in the liver cells are released into the blood, leading to the increase of ALT and AST levels in the sera. Therefore, the levels of serum ALT and AST are the important and sensitive biochemical hallmarks as the assessment of liver function and can indicate early ALD, and their abnormal increase can cause injury and necrosis of liver cells [21]. In the present study, ethanol significantly increased serum ALT and AST levels. Treatment with taraxasterol markedly decreased the increased levels of ALT and AST induced by ethanol, which indicated that taraxasterol could reduce ethanol-induced liver injury by stabilizing hepatocyte membrane. Another important hallmark of alcoholic liver injury is showed by the elevated content of serum and hepatic TG. Under normal conditions, the liver maintains a stable equilibrium state for lipid metabolism. Alcohol can alter the fatty acid composition and cause lipid metabolic disorder in the liver. The level of TG in sera and the liver is related to lipid metabolism, and high level of TG reflects a disruption in lipid metabolism; high level of TG is susceptible and may result in an increase of lipid peroxidation. Alcohol consumption can lead to intrahepatic fatty acid overaccumulation and promote TG synthesis and subsequently steatosis [22]. TG is a sign of fatty degeneration of the liver. According to our study, treatment with taraxasterol obviously decreased the increased TG content induced by ethanol in sera and the liver, which indicated that taraxasterol could effectively improve hepatocyte steatosis and prevent the development of fatty liver. This result is also consistent with the histopathological observation.

Oxidative stress induced by ethanol plays an essential role in the pathogenesis of alcoholic liver disease. Prooxidants and antioxidants in normal biological systems are generally balanced. Both the accumulation of oxidants and the depletion of endogenous antioxidants can cause oxidative stress. Alcohol intake bursts ROS generation and suppresses the antioxidant defense system that is responsible to scavenge the excessive ROS production, leading to oxidative stress in the liver [23]. The excessive ROS reacts with biological macromolecules, proteins, and DNA and leads to hepatocyte damage [24]. In addition, the excessive intake of alcohol can lead to serious oxidative stress in the liver through the production of free radicals and the increase of MDA content, resulting in hepatocyte injury and apoptosis. MDA is the end product of lipid peroxidation and can indirectly reflect the degree of free radical damage on the liver [25]. MDA is also an indicator of the recovery of hepatocytes following drug treatment. In contrast, the antioxidative defense systems GSH and SOD can protect hepatocytes from oxidants, and they can scavenge lipid peroxides and oxygen-free radicals and protect hepatic cells against ROS injury. Alcohol intake increases oxidative stress by inhibiting the level of GSH and the activity of SOD. Thus, GSH level and SOD activity can directly reflect hepatic antioxidant capacity [26]. In our study, treatment with taraxasterol significantly reduced the production of ROS and the level of MDA and increased the level of GSH and the activity of SOD induced by ethanol in the liver tissues. Taking together, the results indicated that taraxasterol could exert antioxidative effects against ethanol-induced oxidative injury by ameliorating oxidative stress and improving the activities of antioxidant enzymes in the liver. These results are consistent with the effects of taraxasterol on primary serum indicators ALT and AST levels.

In order to further study the potential antioxidative stress and oxidant defense mechanisms of taraxasterol on alcoholic liver injury, the expressions of CYP2E1, Nrf2, and HO-I in liver tissues were further investigated. Cytochrome P450 family is the major enzyme systems involved in metabolism in organisms. CYP2E1 is one of the main members of CYP450 family and the most relevant to ALD because of its high inducibility and high catalytic activity [27]. CYP2E1 is mainly present in liver microsomes and plays a critical role in ROS production and liver injury. Acute and chronic alcohol intake increases the activity of CYP2E1, which catalyzes the conversion of ethanol to acetaldehyde and results in excessive ROS production [28]. Excessive ROS can cause oxidative stress in the liver, while suppressing the antioxidant stress defense pathway [3]. In addition, the related protein nuclear factor Nrf2 of the antioxidant stress pathway is an important transcription factor, which induces the encoded detoxification enzymes and antioxidant proteins of the downstream genes in various oxidative stress responses. Under normal states, Nrf2 activity is inhibited by binding to the repressor Kelch-like associated protein 1 (Keap1). Under oxidative stress states, Nrf2 is dissociated from Keap1, transferred into the nucleus, and combined with the transcription factors. Then, the complex combines with the antioxidant response element (ARE) and promotes the transcription of many antioxidant genes such as HO-1 and GSH [21]. Therefore, Nrf2 is considered as a new target for the treatment of alcoholic liver disease. HO-1, a downstream target factor of Nrf2, is a powerful antioxidant that catalyzes the oxidation of protein heme into antioxidant molecules, carbon monoxide, and biliverdin and improves hepatocyte survival. HO-1 can be activated by Nrf2 translocation [29]. In our study, ethanol exposure significantly induced the high expression of CYP2E1 and decreased the expressions of antioxidant genes Nrf2 and HO-1. Treatment with taxarasterol significantly inhibited the ethanol-induced high expression of CYP2E1 and increased the ethanol-induced low expression of Nrf2 and HO-1 in the liver tissue, which indicated that the protective mechanism of taraxasterol on ethanol-induced liver injury might be at least partially attributed to the potential antioxidative stress by suppressing CYP2E1 expression and the enhancement of oxidant defense system by activating Nrf2/HO-I signaling pathway.

In addition to oxidative stress, alcoholic liver injury is mainly characterized by inflammatory response. Inflammation plays a crucial role in the occurrence and development of alcoholic liver disease. Alcohol and its metabolites induce the release of proinflammatory cytokines, which exacerbates liver inflammation and hepatocyte apoptosis [30, 31]. TNF-*α* and IL-6 are two major proinflammatory cytokines in ethanol-induced liver injury, and the persistent expression of TNF-*α* and IL-6 is a notable feature of alcoholic liver injury [32, 33]. In this study, ethanol increased TNF-*α* and IL-6 secretion, and taxarasterol significantly reduced ethanol-induced TNF-*α* and IL-6 secretion, indicating that taraxasterol could protect ethanol-induced liver injury by involving antagonism of proinflammatory cytokines. Many of our previous studies have also shown that taraxasterol has the *in vitro* and *in vivo* anti-inflammatory activity by suppressing the production of various inflammatory cytokines and mediators [15, 17–19, 34]. The present data are consistent with our previous studies.

We further explored the anti-inflammatory mechanism of taraxasterol on alcoholic liver injury. NF-*κ*B signaling pathway is the classical inflammatory signaling pathway. NF-*κ*B is a nuclear transcription factor that regulates the expression of multiple genes in inflammatory response and can be activated by a large number of extracellular stimulating factors. Activation of NF-*κ*B plays an important role in the initiation and progression of alcoholic liver and damages the liver by stimulating the release of inflammatory cytokines. NF-*κ*B is mainly composed of p50 and p65 dimers, which is inactive in cytoplasm and binds to the inhibitory protein I*κ*B under unstimulated state. Once stimulated, the inhibitory protein I*κ*B is phosphorylated and degraded by I*κ*B kinase, and NF-*κ*B is released and transferred to the nucleus and triggers the release of proinflammatory mediators such as TNF-*α* and IL-6 [35–37]. It has been also found that ethanol is catalyzed by ADH as acetaldehyde in the body, acetaldehyde can activate I*κ*B*α* kinase faster and earlier, degrade I*κ*B*α* protein, and increase NF-*κ*B, and TNF-*α* can also increase the expression of NF-*κ*B p65 [38]. In this study, ethanol caused the degradation of I*κ*B*α* protein and increased the expression of NF-*κ*B p65 protein in liver tissues of mice. Treatment with taxarasterol inhibited the degradation of I*κ*B*α* protein and the expression of p65 protein in liver tissues of mice with alcoholic liver injury, which indicated that taraxasterol could inhibit inflammatory response against ethanol-induced liver injury by regulating NF-*κ*B signaling pathway. It is completely consistent with our previous study *in vitro* [15].

## 5. Conclusions

In conclusion, the present study showed that taraxasterol isolated from *Taraxacum* has the protective effects against ethanol-induced liver injury in mice. The protective effects of taraxasterol may be attributed to its ability in reducing the increase of liver index, ALT, AST, TG, and MDA levels, suppressing the reduction of GSH level and SOD activity, inhibiting proinflammatory cytokines TNF-*α* and IL-6 secretion, and improving histopathological alterations in mice with alcoholic liver injury. This potential protective mechanisms may be associated with the reduced oxidative stress, the enhanced oxidant defense systems via the regulation of CYP2E1/Nrf2/HO-I signaling pathway, and the inhibitory inflammatory response via the regulation of NF-*κ*B signaling pathway. These findings imply that taraxasterol may prove to be a useful therapeutic candidate for the prevention and treatment of alcoholic liver disease.

## Data Availability

The data used to support the findings of this study are included within the article.
